# Behaviour change interventions addressing patient antibiotic treatment-seeking behaviour for respiratory tract infections in primary and community care settings: a scoping review

**DOI:** 10.1136/bmjopen-2025-101694

**Published:** 2025-08-05

**Authors:** Anthony Maher, Kevin Roche, Eimear Morrissey, Andrew Murphy, Greg Sheaf, Cristín Ryan, Gerard J Molloy

**Affiliations:** 1 School of Psychology, University of Galway College of Arts Social Sciences and Celtic Studies, Galway, Ireland; 2 University of Galway College of Arts Social Sciences and Celtic Studies, Galway, Ireland; 3 National University of Ireland Galway, Galway, Ireland; 4 University of Dublin Trinity College Library, Dublin, Ireland; 5 School of Pharmacy and Pharmaceutical Sciences, University of Dublin Trinity College, Dublin, Ireland; 6 School of Psychology, University of Galway, Galway, Ireland

**Keywords:** Antibiotics, Behavior, Primary Care, Public health

## Abstract

**Abstract:**

**Objectives:**

This scoping review aimed to map studies on behaviour change interventions that address antibiotic treatment-seeking behaviour for respiratory tract infections in primary and community care settings.

**Design:**

This review is based on the Joanna Briggs Institute guidelines for scoping reviews, guided by the Preferred Reporting Items for Systematic Reviews and Meta-Analyses extension for Scoping Reviews.

**Data sources:**

A literature search in January 2024 and May 2024 was performed across Medline, Embase, CINAHL, PsycINFO, Web of Science Core Collection, Scopus, EThOS and Google Scholar was performed.

**Eligibility criteria:**

Eligible studies described behaviour change interventions in primary and community care settings, published from 2000 onward across all countries.

**Data extraction and synthesis:**

Descriptive data relating to study details and intervention functions were gathered and organised according to the Capability, Opportunity, Motivation and Behaviour change framework in a predeveloped data extraction sheet. Dual data extraction occurred, and inter-rater reliability results are reported (K=0.83).

**Results:**

The scoping review identified 38 eligible studies, which consisted of randomised controlled trials (7/38), cluster randomised controlled trials (6/38), randomised experiments (5/38), cross-sectional studies (5/38), qualitative investigations (5/38) and quasi-experimental designs (4/38). Most interventions focused on educational resources (15/38), digital tools (7/38) and community campaigns (6/38), with fewer targeting decision-making processes (4/38) or psychological drivers of antibiotic-seeking behaviour (3/38). Only one study was conducted in low-income and middle-income countries, and only one separately assessed behaviour change as a measured outcome.

**Conclusions:**

This scoping review highlights a wide range of research methodologies within the topic area. There was some limited evidence of intervention efficacy for antibiotic prescription rates, particularly interventions focused on enhancing knowledge and access to resources. However, more emphasis is needed on standardising outcome measures and evaluating long-term outcomes.

**STRENGTHS AND LIMITATIONS OF THIS STUDY**

- This review includes a wide range of study designs and sources, giving a broad view of the types of behaviour change interventions used to reduce unnecessary antibiotic use for respiratory infections.
- Using the Capability, Opportunity, Motivation and Behaviour framework and established scoping review guidelines helped structure the findings in a way that could be practical for understanding how different interventions are designed and delivered.
- Although the focus was on behaviour change, very few studies actually measured behaviour as an outcome, making it hard to judge how effective these interventions were in changing real-world behaviour.
- Most of the included studies were from high-income countries, which limits how well the findings apply to settings where antibiotics may be used differently or where access to care is more limited.

## Introduction

 Antimicrobial resistance (AMR) poses a significant threat to global public health, jeopardising the effectiveness of treatments for infectious diseases.^1^ Community-acquired respiratory tract infections (RTIs) are often treated with antibiotics. This is both unnecessary and ineffective given their predominant viral aetiology.^2 3^ Addressing behavioural factors linked to prescribers, providers and patients, is critical to curbing further unnecessary use.^4^ Behaviour change interventions (BCIs) are increasingly recognised as a necessary approach for reducing unnecessary antibiotic prescribing, offering tailored strategies to address the complex interplay of individual, societal and systemic determinants of antibiotic use.^4 5^

To effectively evaluate BCIs, an understanding of the underlying behavioural and social dynamics that contribute to antibiotic misuse is required.^6 7^ Antibiotic treatment-seeking behaviour (ATSB) can accelerate the rate of unnecessary antibiotics being provided for RTIs.^8^ ATSB describes the demand for antibiotics by patients or caregivers, ATSB describes the demand for antibiotics by patients or caregivers, often influenced by misperceptions about the effectiveness of antibiotics or expectations for rapid relief of symptoms; such behaviour may carry negative implications, as clinicians can perceive it as challenging their clinical judgement or potentially undermining patient-provider trust.^9 10^ Studies in Italy and Canada indicate that enhancing public awareness regarding necessary antibiotic use can alter patient expectations and reduce unnecessary requests for prescriptions, particularly in paediatric cases.^11 12^ Furthermore, targeted educational interventions aimed at both healthcare providers and patients have shown promise in decreasing unnecessary prescribing rates and improving overall management of RTIs.^13 14^ By fostering a culture of shared decision-making between practitioners and patients, it can not only empower individuals with knowledge, but also cultivate a more responsible approach to antibiotic consumption, mitigating the rise of AMR within communities.^15 16^

While BCIs can enhance shared decision-making by empowering both patients and prescribers, their implementation targeting ATSB can also be tailored to target patient groups in different ways, including educational initiatives, community-based campaigns, shared decision-making tools and fear-based communication strategies.^17^,^19^ The COM-B (Capability, Opportunity, Motivation and Behaviour) model, part of the ‘Behaviour Change Wheel’ framework, identifies Capability, Opportunity and Motivation as the key factors influencing behaviour change.^20^ By addressing these elements, BCIs can be more systematically designed to encourage appropriate antibiotic use. These interventions aim to improve knowledge, reshape social norms and realign motivations to support rational antibiotic use.^21 22^ Despite the diversity of some of these known interventions, a lack of consistency in their design, delivery and evaluation presents challenges to understanding their overall breadth and effectiveness.^23^ This necessitates a systematic examination of BCIs breadth and extent, particularly those that target key behavioural domains, such as psychological capability, opportunity and motivation as described in the COM-B model.^20^

Scoping reviews are well-suited to explore areas with diverse evidence, making them an appropriate methodology for synthesising the extent and types of BCIs addressing ATSB for RTIs. By integrating a wide range of study designs and patient populations, this scoping review provides a comprehensive understanding of intervention strategies and their potential impact on ATSB. A preliminary search for existing reviews confirmed the absence of comprehensive syntheses examining BCIs aimed at non-healthcare professionals (non-HCPs) for ATSB in primary and community care contexts. The primary aim of this review was to synthesise evidence on BCIs targeting ATSB for RTIs in primary and community care settings. Specifically, the review investigated the content, context and mode of delivery of these interventions, their alignment with theoretical frameworks (if any), and their reported measured outcomes.

### Review question

This review aimed to identify the scope and nature of evidence regarding BCIs that target ATSB for RTIs within primary and community care settings.

The research questions that outlined the aim of the review are:

- Which BCIs have been implemented and/or assessed to diminish ATSB for RTIs?
- What characteristics of BCIs can be identified by aligning them with the COM-B model found within the ‘Behaviour Change Wheel’ framework?
- What behaviour change theories are integrated within the interventions aimed at reducing ATSB for RTIs, if any?
- What gaps currently exist in the literature that requires attention in future research regarding BCIs?

### Inclusion criteria

#### Types of participants

Eligible studies concentrated on patients and/or caregivers within primary and community care, who present with or discuss symptoms typically associated with an RTI, as delineated by the International Classification of Diseases (ICD)-10 RTI symptom criteria.^24^

#### Concept

Relevant studies concerning ATSB published in the English language from any country that focus on primary and community care will be included.

#### Types of sources of evidence

Published and unpublished qualitative, quantitative, mixed-methods and descriptive studies, written since 2000 and written in English were included due to pragmatic reasons. No limitations were placed on the study location.

## Methods

### Search strategy

The following electronic databases were searched: Medline, Embase, CINAHL, PsycINFO, Web of Science Core Collection, Scopus, EThOS and Google Scholar.

Initial search terms were decided with input from advisory panel members with research and clinical experience in primary and community care (AMu and CR). In consideration of the subject heading attributes of the search fields, a tailored search strategy was developed for each database in consultation with a specialist librarian (GS) (see online supplemental I). A variation of terminology describing BCIs targeting unnecessary antibiotic for RTIs is recognised, and so, a broad range of terms covering BCIs was included.

Data were sourced using an academic database search, as well as manually searching citation lists of all included studies at the full-text stage. Databases were searched at two time points—January 2024 and May 2024. Search results were saved after removing duplicate literature using Covidence online platform and its associated reference management software.

### Source of evidence screening and selection

Two reviewers (AMa and KR) independently completed title and abstract screening, and full-text screening of all selected studies on Covidence database. Disagreements that arose on a study during each screening stage were resolved through a third member of the review team (EM/GJM).

### Data extraction

After the full-text studies were independently selected, two reviewers (AMa and KR) completed dual data extraction on 20% of studies using a data extraction form developed in accordance with the JBI guidelines and the study protocol (see online supplemental II).^25 26^ Inter-rater reliability results were calculated according to kappa statistics. The remaining data extraction was completed by one reviewer (AMa). The following information was extracted for each included study:

1. Title.
2. Author.
3. Year.
4. Location.
5. Sample size.
6. Study design.
7. Intervention description.
8. Behavioural target.
9. COM-B framework component.
10. Intervention function.
11. Mode of delivery.
12. Theoretical basis.
13. Outcome.
14. Effectiveness.

### Analysis and presentation of results

Data are presented according to the Preferred Reporting Items for Systematic Reviews and Meta-Analyses extension for Scoping Reviews and the scoping reviews protocol.^26 27^ A narrative synthesis of the findings according to the guidelines was performed due to a varied extent of study design and measured outcomes.^28^

Analysis of the data included subjectively assessing each study’s intervention to determine its alignment with one or more of the COM-B components. In cases where interventions spanned multiple COM-B components, data were coded to all relevant domains.

Through an iterative process, themes and subthemes were proposed for each COM-B model component relevant to the context. Focusing on descriptions of intervention components and their intended mechanisms of action, data were then mapped to the identified themes and subthemes. This process was used to categorise and understand how BCIs targeting ATSB for RTIs aligned with a commonly used theoretical framework that can be incorporated into intervention design, interpretation and analysis.

Data were also extracted by examining intervention descriptions. Discussions between reviewers (AMa and KR) resolved discrepancies during the dual data extrication process as needed.

### Patient and public involvement

None.

## Findings/results

### Search results

Of the 7857 studies identified during the preliminary search, 2178 duplicates were removed. After reviewing the titles and abstracts of 5674 studies, 5458 studies were excluded due to the predetermined exclusion criteria. After reviewing the full-text studies, 38 studies were selected for data extraction (see online supplemental III). These studies were manually searched through their citations; however, no further studies were identified from this process. Inter-rater reliability measured as a kappa statistic value was 0.83 for data extraction.

### Inclusion of sources of evidence

In terms of geographical diversity, the interventions (see online supplemental IV) were implemented across multiple nations including the UK,^1718 29^,^38^ the USA,^39^,^48^ Australia,^49 50^ Canada,^51 52^ Germany,^53 54^ the Netherlands,^55 56^ Cyprus,^57^ Indonesia,^58^ Singapore,^59^ Malaysia,^60^ Spain,^61^ Hong Kong,^62^ Denmark^63^ and one study assessing various low-income and middle-income countries (LMICs).^64^ The methodological approaches adopted consisted of randomised controlled trials,^17 18 35 36 48 54 59^ cluster randomised controlled trails,^30 34 40 41 46 56^ randomised experiments,^33 37 38 47 53^ mixed methods,^29 32 52^ qualitative investigations,^30 54 55 61 64^ cross sectional studies,^39 44 45 50 58^ preintervention and postintervention studies,^43 63^ interrupted time-series analysis^51 57^ and quasi-experimental design.^29 42 60 62^

The sample sizes displayed considerable variability, ranging from smaller qualitative assessments involving 18–45 participants to multisite trials accommodating as many as 1500 participants. The populations that were the focus of these studies encompassed parents and guardians, individuals suffering from RTIs and general members of the community. The durations for the execution and data gathering were heterogeneous, with some investigations conducting longitudinal analyses over several years, while others concentrated on more abbreviated intervention periods, such as daily or weekly.^38 43 51 53^

### Review findings

#### Intervention types

The interventions implemented had considerable heterogeneity, encompassing a spectrum from once-off educational resources to sustained multilevel public health initiatives. Many interventions targeted parents, such as interactive booklets^30 56^ and tailored informational materials^48 55^, which aimed to improve understanding of symptom management and appropriate antibiotic use. Community-focused efforts, including national campaigns like ‘Using Antibiotics Wisely’ in Canada^51^ and programmes in Cyprus^57^ and Australia,^50^ were designed to raise public awareness and encourage judicious use of antibiotics for RTIs. Fear-based messaging^37^ and community-specific materials^54^ were personalised or culturally tailored.

Brief digital^62^ and multimedia strategies, such as animated films^17^ and online interventions,^38^ were designed to enhance patient education and broaden accessibility. Tools aimed at shared decision-making and improved clinical communication techniques underpinned interactions between patients and clinicians. Clinical communication improvements,^39^ such as sharing information leaflets during consultations,^29 34^ sought to enhance both patient understanding and trust in non-antibiotic management strategies.

#### Target groups

Individuals across all age demographics suffering from RTIs were addressed, most notably in research involving collaborative decision-making frameworks.^49 51^ Parents of children exhibiting symptoms related or diagnosed with RTIs constituted a primary focus.^3039 44 45 53 55^,^57 63 65^ Digital platforms appear to expand the scope to engage patients and the broader public with tailored content,^38 62^ while vulnerable populations, such as older adults,^42^ were specifically targeted through age-appropriate educational programmes. Certain studies addressed culturally specific populations, like Turkish immigrants in Germany.^54^ Wider public demographics were engaged through national initiatives, such as those executed in Australia and Malaysia, which aimed to transform community standards concerning unnecessary antibiotic use.^50 60^

#### Outcome measures and effectiveness

The diversity of outcome measures was evident, reflecting the studies’ geographical contexts, sample sizes and study designs; however, many were centred on the objective of minimising unnecessary antibiotic use. Two out of 38 studies evaluated shifts in knowledge and awareness pertaining to antibiotic resistance and necessary use.^17 61^ Digital tools and media, including animated films and computerised modules, improved public knowledge about AMR by 35%–50% and reduced inappropriate behaviours, such as seeking, requesting or using antibiotics for viral or self-limiting conditions, by up to 30%.^17 46 62^ Behavioural outcomes in four studies encompassed reductions in antibiotic prescriptions (up to 6% increase in participants intending not to ask for antibiotics) and instances of self-medication (low desire for antibiotics measured as 22%–49% posteducational module intervention).^17 38 46 52^

Parental anticipations regarding antibiotic prescriptions were assessed in studies following educational initiatives, where reductions ranged from 20% to 28%.^39 45 55 63^ Wider public health initiatives, including community-based campaigns, led to reductions in antibiotic prescriptions of 20%–40%, reflecting the efficacy of population-level strategies.^49 51^ Similarly, tools like the TARGET leaflet and interactive booklets proved effective in reducing reconsultation rates (20%–32%) and antibiotic prescribing (15%–25%), particularly for RTIs.^29 30 56^

#### Behavioural targets

The studies highlighted target various behavioural changes regarding antibiotic use across different populations. Some focus on general public awareness and behavioural shifts regarding antibiotic use for RTIs, where efforts aim to reduce unnecessary antibiotic demand through education and media campaigns.^50 51^ In contrast, parental education emerges as a key behavioural target in numerous studies, focusing on reducing antibiotic requests for children with RTIs.^48 55 57^ Furthermore, several studies target patient decision-making and expectations directly, as they aim to reduce unnecessary antibiotic use through digital modules, and fear-based messaging focused on AMR.^36 46 62^

#### COM-B framework related components

##### Capability: psychological understanding

Most interventions aimed to enhance psychological capability by improving understanding of medication use and illness management. For instance, educational banners in Indonesia and digital interventions in Hong Kong focused on enhancing knowledge about appropriate antibiotic use and AMR.^58 62^ Similarly, interactive booklets in the Netherlands and the UK provided parents with guidance on managing childhood infections, reducing the reliance on antibiotics.^55 56^ In the USA, consultations integrated information on antibiotic stewardship, educating patients about the limited role of antibiotics in treating viral infections like acute bronchitis.^47^ Tailored interventions, such as culturally relevant materials in Germany, further increased understanding by addressing language and cultural barriers.^54^

##### Opportunity: physical and social influences

Physical opportunity was provided by access to educational resources, such as leaflets, booklets and online campaigns, making information readily available. In Cyprus and Canada, community-based initiatives leveraged social influence to shape public norms and encourage judicious antibiotic use.^51 57^ Interventions often involved access to consultations with HCPs, allowing patients to discuss symptoms and understand appropriate treatments.^34^,^3657^ Programmes like the TAP initiative targeted cultural contexts, emphasising community engagement to foster supportive environments for reduced antibiotic use.^43^

##### Motivation: reflective and automatic components

Reflective motivation was a key focus, with many interventions aiming to change beliefs and alter patient expectations regarding antibiotics. Campaigns like ‘Using Antibiotics Wisely’ in Canada and fear-based messaging in the UK emphasised the risks of misuse, fostering belief changes.^37 51^ Automatic motivation was targeted through strategies to build confidence in managing illnesses without antibiotics, such as providing emotional reassurance via booklets and consultations.^36 44 52^ Habit formation was also encouraged, as seen in US well-child care visits where patients were educated on non-antibiotic management strategies.^41 48^

## Discussion and conclusions

This scoping review explored BCIs aimed at addressing ATSB concerning RTIs within primary and community care settings. Employing the COM-B model, the interventions were systematically classified by the authors according to their potential mechanisms of action, thereby facilitating a comprehensive understanding of how these interventions tackle the behavioural determinants of unnecessary antibiotic use.^20^ The findings reveal a spectrum of study design, intervention strategies, their differing degrees of efficacy and the specific populations they are designed to serve. This review contextualises the results within the broader literature and assesses the strengths and limitations inherent in the included studies.^66^,^68^

Public health campaigns, such as those implemented in Australia and Canada, successfully elevated public awareness and transformed community norms concerning antibiotic utilisation.^50 51^ Comparable successes have been documented in other contexts, with initiatives such as the European Antibiotic Awareness Day fostering enhanced public comprehension of AMR and diminishing antibiotic demand.^69^ At the individual level, interventions using educational resources, including interactive booklets and digital platforms, exhibited a potential for enhancing knowledge and mitigating unnecessary antibiotic use.^30 56^ For instance, a study conducted in the UK indicated that the distribution of educational booklets in primary and community care settings led to a decrease in patient consultations and antibiotic prescriptions.^70^ Such strategies underscore the importance of contextually tailored interventions that actively engage patients.

The review further established that BCIs were potentially effective in curtailing unnecessary antibiotic usage, although the outcomes exhibited variability contingent on context and population characteristics.^50 54 60 61 64^ Shared decision-making tools and educational interventions empowered individuals to make informed decisions.^29 58^ This observation aligns with a meta-analysis conducted, which evidenced that shared decision-making diminishes unnecessary antibiotic prescribing in primary and community care settings.^19^ Nonetheless, the efficacy of fear-based messaging, which yielded positive results, also deserves scrutiny.^37^ While this finding is consistent with other evidence underscoring the influence of emotion on behaviour change,^71^,^73^ ethical considerations persist regarding the potential for inducing anxiety or distress among patients.

Interventions predicated on the COM-B framework offered valuable insights into the psychological, social and structural determinants influencing behaviour. Most interventions focused on enhancing psychological capability through the improvement of knowledge and skills.^33 58 63^ This observation is congruent with studies indicating that augmenting patients’ comprehension of antibiotic use and resistance constitutes a critical preliminary step in facilitating behaviour change.^74 75^ For example, interventions such as the TARGET leaflet,^29^ which enhanced patient understanding of RTI management, reflect the successes of analogous tools employed in global AMR awareness campaigns. Additionally, social and physical opportunities were strategically harnessed in the reviewed interventions. Community educational initiatives established platforms for nurturing supportive environments and improving access to credible information, akin to strategies proven effective in resource-limited settings.^40 50 51 66 76 77^

However, from the included studies, reflective motivation is comparatively underused in relation to capability and opportunity, notwithstanding evidence indicating its critical role in the maintenance of behaviour change.^78^,^80^ Interventions designed to modify patient perceptions regarding the necessity of antibiotics, such as fear-inducing or persuasive communication strategies, have exhibited measured efficacy in other contexts.^80^,^83^ A systematic review on nudge interventions demonstrated that persuasive messaging aimed at addressing parental apprehensions about antibiotic use effectively diminished the occurrence of unnecessary prescriptions within paediatric care.^84^ Likewise, interventions that enhance self-management confidence have proven successful in curtailing antibiotic dependence for minor ailments.^37 85 86^ These findings underscore the imperative to harmonise educational methodologies with tactics that directly engage motivational elements to maximise intervention efficacy.

Primarily, this scoping review implemented a search strategy and stringent methodological framework, conforming to the guidelines established by JBI, thereby ensuring the inclusion of a wide array of interventions across varied contexts.^25^ The adoption of the COM-B framework facilitated a systematic approach for categorising interventions, thus permitting a more nuanced comprehension of the behavioural mechanisms that underpin their effectiveness. Furthermore, the synthesis of evidence drawn from diverse study designs, including randomised controlled trials and qualitative assessments, allowed for a comprehensive evaluation of intervention efficacy.

Notwithstanding these strengths, several limitations merit attention. The subjective nature inherent in the mapping of interventions to COM-B components may introduce bias, as disparate reviewers may interpret intervention mechanisms in divergent manners. While efforts aimed at ensuring consistency, such as collaborative deliberations, partially mitigated this risk, the fundamental subjectivity remains a recognised limitation. The variability among the included studies regarding design, population and outcome measures presented challenges for comparative analysis and synthesis. For instance, whereas certain studies quantified antibiotic prescriptions as outcomes of interest, others concentrated on knowledge or attitudinal modifications, complicating the derivation of overarching conclusions.

Another limitation pertains to the insufficient representation of interventions within LMICs, where impediments to behaviour modification, such as restricted healthcare access, are notably more acute. This under-representation aligns with conclusions drawn from other reviews, which highlight a scarcity of evidence originating from LMICs concerning AMR-related interventions.^75 87^ However, the exclusion of non-English studies and the limitation to publications pre-2000 may have resulted in the omission of pertinent evidence, particularly from regions with established initiatives aimed at combating unnecessary antibiotic use.

Additionally, a limited number of studies assessed the long-term sustainability of interventions, raising concerns regarding the persistence of behaviour change over time. This observation corresponds with broader apprehensions within behaviour change research, where immediate outcomes frequently overshadow long-term ramifications.^20 88 89^

The majority of included studies in this review were carried out in high-income countries (HICs) where the prevailing population health challenges and health services limitations are very different to LMICs. For example, there are typically significantly different patterns of infectious diseases and associated acute illnesses, for example, diarrhoea and fever, and there can be a shortage or complete absence of trained prescribers of antibiotics and public health resourcing.^90 91^ In addition, there can be limitations in population health literacy and a diversity of informal health communication channels, which mean that the causes and communication solutions to unnecessary use of antibiotics are likely to be a different form than HIC contexts.^92^,^94^ Therefore, BCIs need to factor in the appreciable differences that can exist between HICs and LMICs in terms of available resources and personnel needed to implement any BCIs to reduce inappropriate use of antibiotics.

### Conclusions and recommendations

In conclusion, this scoping review illustrates the breadth and measured significance of BCIs in ameliorating ATSB for RTIs within primary and community care settings.

The results indicate that interventions aimed at enhancing psychological capability and physical opportunity, such as educational resources and community involvement, are efficacious in mitigating unnecessary antibiotic use when measured at specific outcomes. Nevertheless, reflective motivation, which is crucial for fostering enduring behavioural change, remains insufficiently leveraged.

Subsequent research could prioritise interventions that address motivational components, especially in underrepresented contexts such as LMICs. Furthermore, investigating the long-term viability of these interventions will be essential for ensuring sustained outcomes. By addressing these deficiencies, stakeholders could formulate more effective, evidence-based approaches to combat antibiotic resistance.

## Supplementary material

10.1136/bmjopen-2025-101694 online supplemental file 1

10.1136/bmjopen-2025-101694 online supplemental file 2

10.1136/bmjopen-2025-101694 online supplemental file 3

10.1136/bmjopen-2025-101694 online supplemental file 4

## Data Availability

Data sharing not applicable as no datasets generated and/or analysed for this study.
