# Supplementary material for: Behaviour change interventions addressing patient antibiotic treatment-seeking behaviour for respiratory tract infections in primary and community care settings: a scoping review
Source: BMJ Open. 2025 Aug 5;15(8):e101694. doi: 10.1136/bmjopen-2025-101694 (PMC12336482; doi:10.1136/bmjopen-2025-101694)
Supplement: online supplemental file 1 [file bmjopen-15-8-s001.docx]

Master Search Logic from Web of Science Database: (((Respiratory OR chest) NEAR/2 (disease* OR infection*)) OR "RTI*" OR Bronchitis OR "Common cold*" OR Croup OR Epiglottitis OR Flu OR Influenza* OR Laryngitis OR Laryngotracheitis OR Otalgia OR Pharyngitis OR Pleurisy OR Pneumo* OR Rhinitis OR Sinusitis OR Tonsillitis OR Breathless* OR Congestion OR Cough* OR Earache* OR Headache* OR "High temperature*" OR "Muscle ache*" OR "Runny nose*" OR Sneez* OR "Sore throat*" OR Wheez*)

AND

(((Family OR General) NEAR/2 (doctor* OR physician* OR pract*)) OR ((Community OR Primary) NEAR/2 (care OR center* OR centre* OR healthcare)) OR Ambulatory OR Pharmac* OR (Home NEAR/2 visit*))

AND

((antibiotic* OR "anti biotic*" OR antimicrob* OR "anti microb*") NEAR/5 (resistan* OR prescrib*))

AND

(accept* OR access* OR approach* OR availab* OR behav* OR chang* OR communicat* OR convenien* OR decision* OR engag* OR guid* OR influen* OR intention* OR interven* OR motiv* OR nudg* OR persua* OR plan* OR polic* OR procedure* OR program* OR strateg* OR support* OR train* OR uptake*)

MEDLINE (PubMed): MeSH terms

"Respiratory Tract Infections", "Bronchitis", "Influenza, Human", "Pneumonia", "Sinusitis", "Pharyngitis", "Tonsillitis", "Laryngitis", "Rhinitis", "Otitis Media", "Croup", "Physicians, Family", "General Practitioners", "Primary Health Care", "Community Health Services", "Ambulatory Care", "Pharmacies", "Anti-Bacterial Agents", "Drug Resistance, Bacterial", "Drug Prescriptions", "Drug Utilization", "Health Behavior", "Behavioral Medicine", "Patient Participation", "Patient Education as Topic", "Health Communication", "Decision Making"

EMBASE: Emtree terms

"respiratory tract infection", "bronchitis", "influenza", "pneumonia", "sinusitis", "pharyngitis", "tonsillitis", "laryngitis", "rhinitis", "otitis media", "croup", "general practitioner", "family physician", "primary health care", "community care", "ambulatory care", "pharmacy", "antibiotic agent", "bacterial resistance", "drug prescription", "drug utilization", "health behavior", "behavioral medicine", "patient participation", "patient education", "health communication", "decision making".

CINAHL (via EBSCOhost): CINAHL headings

"Respiratory Tract Infections", "Bronchitis", "Influenza", "Pneumonia", "Sinusitis", "Pharyngitis", "Tonsillitis", "Laryngitis", "Rhinitis", "Otitis Media", "Croup", "Family Practice", "General Practice", "Primary Health Care", "Community Health Services", "Ambulatory Care", "Pharmacy Services", "Antibiotic Agents", "Drug Resistance", "Drug Prescribing", "Health Behavior", "Patient Participation", "Patient Education", "Health Communication", "Decision Making"

PsycINFO: APA Thesaurus terms

"Antibiotic Resistance", "Antibiotic Prescribing", "Behavior Change", "Behavioral Intentions", "Clinical Decision Making", "Communication", "Community Health", "Cough", "Family Medicine", "Health Behavior", "Health Education", "Influenza", "Patient Attitudes", "Patient Education", "Primary Care", "Respiratory Tract Infections", "Sinusitis", "Tonsillitis", "Treatment Adherence"

Scopus: Scopus’s advanced search options for terms combined with Boolean operators

"Antibiotic Resistance", "Antibiotic Prescribing", "Behavior Change", "Behavioral Intention", "Clinical Decision Making", "Communication", "Community Health", "Cough", "Family Medicine", "Health Behavior", "Health Education", "Influenza", "Patient Attitudes", "Patient Education", "Primary Care", "Respiratory Tract Infections", "Sinusitis", "Tonsillitis", "Treatment Adherence"

EThOS: "antibiotic resistance" AND "respiratory tract infections" AND "primary care" AND (behaviour OR intervention OR communication OR access OR prescribing)

Google Scholar: "antibiotic resistance" AND "respiratory tract infections" AND "primary care" AND (behaviour OR intervention OR communication OR access OR prescribing)
