# Supplementary material for: Behaviour change interventions addressing patient antibiotic treatment-seeking behaviour for respiratory tract infections in primary and community care settings: a scoping review
Source: BMJ Open. 2025 Aug 5;15(8):e101694. doi: 10.1136/bmjopen-2025-101694 (PMC12336482; doi:10.1136/bmjopen-2025-101694)
Supplement: online supplemental file 2 [file bmjopen-15-8-s002.docx]

| Title | Author(s), year | Location | Sample size | Study design | Intervention Description | Behavioural target | COM-B Framework Components - Capability | COM-B Framework Components - Opportunity | COM-B Framework Components - Motivation | Intervention functions | Mode of delivery | Theoretical basis | Outcome | Effectiveness |
| --- | --- | --- | --- | --- | --- | --- | --- | --- | --- | --- | --- | --- | --- | --- |
|  |  |  |  |  |  |  |  |  |  |  |  |  |  |  |
|  |  |  |  |  |  |  |  |  |  |  |  |  |  |  |
|  |  |  |  |  |  |  |  |  |  |  |  |  |  |  |
