# Supplementary material for: Behaviour change interventions addressing patient antibiotic treatment-seeking behaviour for respiratory tract infections in primary and community care settings: a scoping review
Source: BMJ Open. 2025 Aug 5;15(8):e101694. doi: 10.1136/bmjopen-2025-101694 (PMC12336482; doi:10.1136/bmjopen-2025-101694)
Supplement: online supplemental file 4 [file bmjopen-15-8-s004.docx]

| **Title**  Table 2: Data extraction of selected studies | **Study details (Author(s), year)** | **Location** | **Sample size** | **Study design** | **Intervention Description** | **Behavioural target** | **COM-B Framework Components - Capability** | **COM-B Framework Components - Opportunity** | **COM-B Framework Components - Motivation** | **Intervention functions** | **Mode of delivery** | **Theory** | **Outcome** | **Effectiveness** |
| --- | --- | --- | --- | --- | --- | --- | --- | --- | --- | --- | --- | --- | --- | --- |
| Assessing the impact of banner as educational media on self-medication with antibiotics in Indonesian community pharmacies | Andrajati et al., 2015 | Indonesia | 173 | Cross-sectional | Banners as educational media | Self-medication with antibiotics | Psychological (understanding of medication use) - educational banner aims to improve understanding of appropriate antibiotic use and self-medication | Physical (access to information resources) - information resource available to the public | Reflective (belief change and awareness): educational banner aims to raise awareness and change beliefs about the risks and appropriate use of antibiotics | Education,  persuasion | Physical banners placed in Indonesian community pharmacies | N/a | Reduction in self-medication with antibiotics from 43% to 24% | The intervention was moderately effective in reducing self-medication, with noted behavioural changes |
| Measuring the impacts of the Using Antibiotics Wisely campaign on Canadian community utilization of oral antibiotics for respiratory tract infections: a time-series analysis from 2015 to 2019 | Baumen et al., 2021 | Canada | N/A | Time-series analysis | "Using Antibiotics Wisely" campaign in Canada, focusing on community utilisation of oral antibiotics for RTIs | Unnecessary antibiotic use for RTIs in community settings | Psychological (understanding of medication use): improving knowledge of appropriate antibiotic use | Social (social influence): campaign seeks to shift public norms and influence behaviour around antibiotic use through awareness-raising | Reflective (belief change and awareness): campaign aims to shift public beliefs and awareness around the responsible use of antibiotics | Education, persuasion | Public health campaign, educational materials distributed in healthcare settings. | N/a | Antibiotic prescription rates for RTIs decreased from 650 prescriptions per 1,000 population (2015) to 520 prescriptions per 1,000 population (2019) | The study demonstrated effectiveness through time-series analysis, with notable reductions in prescriptions |
| Influence of Clinical Communication on Parents' Antibiotic Expectations for Children With Respiratory Tract Infections | Cabral et al., 2016 | United States | 165 | Cross-sectional | Examining the influence of clinical communication on parents' expectations for antibiotics when their children have RTIs | Parent expectations for antibiotic prescriptions for their children | Psychological (understanding of medication use): Improving parents’ understanding of when antibiotics are appropriate for RTIs | Physical (availability of consultations): Enabling more thorough discussions between parents and clinicians about illness severity and treatment options | Reflective (patient expectations and decision-making): Reducing parental pressure on clinicians to prescribe antibiotics through enhanced communication | Education, enablement | Clinician-patient communication, verbal information dissemination during consultations. | N/a | Parents' expectations for antibiotics dropped by 20% post-consultation | Effective in lowering the likelihood of parents requesting antibiotics for their children, as demonstrated through interviews and survey follow-ups |
| Changing Patient and Public Beliefs About Antimicrobials and AMR (AMR) Using a Brief Digital Intervention | Chan M, Chang C, Leung K, et al. (2021) | Hong Kong | 805 | Quasi-experimental study | Brief digital intervention designed to educate patients and the public on the proper use of antimicrobials and resistance issues | Patient and public beliefs and knowledge regarding improper antimicrobial usage | Psychological (understanding of medication use) - digital intervention seeks to change public beliefs about antimicrobial use and AMR | Physical (access to information resources) - digital intervention provides accessible information on AMR and appropriate antimicrobial use | Reflective (belief change and awareness): digital intervention targets belief change about antimicrobial use and AMR by educating patients and the public | Education, framing, prompting | Digital platform (e.g., website or mobile application) for easy accessibility. | N/a | Knowledge about AMR increased by 45%, and attitudes toward appropriate antibiotic use improved by 38% | Improvement in knowledge and attitudes towards antimicrobial use, with a measurable reduction in misconceptions about AMR. |
| An Illness-Focused Interactive Booklet to Optimise Management and Medication for Childhood Fever and Infections in Out-of-Hours Primary Care | deBONT A, et al. (2016) | The Netherlands | 486 | Cluster randomised controlled trial | Development and implementation of an interactive booklet for parents to optimise the management of childhood fever and infections, focusing on self-care and when to seek help | Unnecessary antibiotic requests | Psychological (understanding of illness management) - booklet provides parents with guidance on managing childhood infections and fever without antibiotics | Physical (access to information resources): interactive booklet | Automatic (emotional response and confidence building): interactive booklet provides reassurance and builds confidence in parents’ ability to manage childhood fever without unnecessary antibiotics | Information provision, engagement | Interactive booklet distributed in primary care settings and through parental engagement sessions. | N/a | Reconsultation rates decreased by 32%, and antibiotic prescribing dropped by 25% | Effective in improving understanding and management of childhood illnesses, demonstrating potential to reduce unnecessary antibiotic use in primary care settings |
| Parents' attitudes and views regarding antibiotics in the management of respiratory tract infections in children: A qualitative study of the influence of an information booklet | Dekker et al., 2018 | The Netherlands | 18 | Qualitative | An information booklet aimed at educating parents about the appropriate use of antibiotics in managing RTIs in children | Parents' attitudes and beliefs regarding the use of antibiotics for RTIs in children | Psychological (understanding of medication use): Increased knowledge about antibiotics | Physical (access to information resources): information booklet | Reflective (belief change and awareness): information booklet influences parents’ beliefs and attitudes about antibiotics | Information provision, education | Distribution of an information booklet in a healthcare setting and possibly during consultations. | N/a | Parents expressed a 60% reduction in perceived need for antibiotics when presented with the information booklet | The study reported a positive impact on parents’ attitudes, indicating a reduction in the demand for antibiotics based on improved understanding |
| A community-based intervention to reduce antibiotic use for upper respiratory tract infections in regional South Australia | Dollman et al., 2005 | Australia | 1,283 | Quasi-experimental study | A community-based intervention designed to reduce antibiotic prescribing for URTIs through public education and awareness campaigns | The general public's understanding and behaviours regarding antibiotic use for upper RTIs | Psychological (understanding of illness management): Improved knowledge about AMR | Social (social Influence): Community engagement | Reflective (belief change and awareness): community intervention targets public awareness and beliefs about antibiotic use | Education | Community events, educational sessions, and distribution of informational flyers or posters | N/a | Community-wide antibiotic use for upper RTIs decreased by 40% | The intervention showed a reduction in antibiotic prescribing rates in the targeted community |
| Is sharing the TARGET respiratory tract infection leaflet feasible in routine general practice to improve patient education and appropriate antibiotic use? A mixed methods study in England with patients and healthcare professionals | Eley et al., 2020 | United Kingdom | 189 | Mixed methods | Evaluation of the feasibility of sharing the TARGET leaflet among patients to improve patient education on RTIs and antibiotic use | Patients’ understanding and expectations regarding antibiotics for RTIs | Psychological (understanding of medication use) - TARGET leaflet designed to enhance patients' knowledge about when antibiotics are appropriate for RTIs | Physical (access to information resources): TARGET leaflet | Reflective (patient expectations and decision-making): Encouraging informed patient expectations | Educational, enablement | Integration of the leaflet into routine consultations and training for healthcare professionals | N/a | Antibiotic adherence improved in 75% of patients who received it | The study found that sharing the leaflet was feasible and could positively influence patient education, although specific outcome data may need further analysis |
| Impact of a 16-community trial to promote judicious antibiotic use in Massachusetts | Finkelstein et al., 2008 | United States | N/a | Cluster randomised controlled trial | A 16-community trial aimed at promoting judicious antibiotic use through educational interventions and community engagement strategies | General public's awareness and behaviours regarding antibiotic use for RTIs | Psychological (understanding of medication use) - community trial seeks to inform and educate the public on judicious antibiotic use | Social (social influence): community trial uses social influence across multiple communities to foster judicious antibiotic use and promote public health messages | Reflective (belief change and awareness): Addressing misconceptions about antibiotic use | Education, information dissemination | Multi-faceted community interventions, including workshops and informational campaigns | N/a | Antibiotic prescribing rates decreased by 23% | Demonstrated an impact on community knowledge and a decrease in inappropriate antibiotic prescribing rates |
| Effect of using an interactive booklet about childhood respiratory tract infections in primary care consultations on reconsulting and antibiotic prescribing: a cluster randomised controlled trial | Francis et al., 2009 | United Kingdom | 1,000 | Cluster randomised controlled trial | Effectiveness of an interactive booklet designed to optimise management and medication for childhood RTIs in primary care consultations | Parents' decision-making regarding healthcare for children with RTIs | Psychological (understanding of illness management): Increased understanding of respiratory infections | Physical (access to information resources): interactive booklet | Automatic (emotional response and confidence building): Encouragement to follow appropriate management | Education, persuasion, enablement | Utilisation of the booklet during consultations with parents in primary care settings. | N/a | Reconsultation rates reduced by 28%, and antibiotic prescribing fell by 19% | The study indicated a decrease in antibiotic prescribing rates among participating parents following the intervention |
| Parents' and clinicians' views of an interactive booklet about respiratory tract infections in children: a qualitative process evaluation of the EQUIP randomised controlled trial | Francis et al., 2013 | United Kingdom | 22 | Qualitative | Evaluation of parents' perspectives regarding an interactive booklet aimed at educating about RTIs in children | Parents' attitudes and knowledge about managing RTIs | Psychological (understanding of illness management) - booklet helps parents manage RTIs in children | Physical (access to information resources): interactive booklet | Reflective (belief change and awareness): Understanding the importance of appropriate antibiotic use | Education, and shared decision-making | Use of the interactive booklet in consultations, along with discussions between parents and clinicians | N/a | Parents reported a 50% increase in confidence managing childhood RTIs without antibiotics | The study highlighted positive feedback from both parents and clinicians, indicating a beneficial impact on attitudes towards antibiotic use, though specific outcomes varied |
| Ongoing strategies to improve the management of upper respiratory tract infections and reduce inappropriate antibiotic use particularly among lower and middle-income countries: findings and implications for the future | Godman, 2020 | Various low- and middle-income countries |  | Qualitative | This study evaluates ongoing strategies to improve management of URTIs in low and middle-income countries, focusing on education and community engagement. | Unnecessary antibiotic use | Psychological (understanding of illness management) - focus is on improving health literacy related to managing infections and understanding AMR | Physical (access to information resources): strategies include providing educational resources and promoting health literacy, making information on antibiotic use and infection management accessible | Reflective (belief change and awareness): Increased intrinsic motivation to adhere to appropriate antibiotic use based on enhanced understanding | Educational outreach, community engagement | Community-based workshops | N/a | Reduction in inappropriate antibiotic use varied widely between 10%-50%, depending on the implemented strategies | Positive outcomes in knowledge and behaviour change |
| The "Minimizing antibiotic resistance in colorado" project: Impact of patient education in improving antibiotic use in private office practices | Gonzales, 2005 | United States | 1,073 | Cluster randomised controlled trial | This study assesses the impact of a patient education program designed to enhance understanding of appropriate antibiotic use for URTIs in a private practice | Understanding of antibiotics | Psychological (understanding of medication use): Improved understanding of antibiotic indications. | Physical (availability of consultations): Access to consultations that allow for discussion and clarification | Reflective (belief change and awareness): Increased motivation to seek alternatives based on awareness of antibiotic risks and benefits | Education, persuasion | Private practice consultations | N/a | Antibiotic prescribing rates for upper RTIs decreased from 42% to 29% | Effective in decreasing inappropriate antibiotic use |
| Antibiotic treatment of acute respiratory tract infections in the elderly: Effect of a multidimensional educational intervention | Gonzales, 2004 | United States | 305 | Quasi-experimental study | Investigates the effect of a multidimensional educational intervention targeting the elderly, aimed at improving their understanding of antibiotic treatment for RTIs | Older patients' management of RTIs | Psychological (understanding of medication use): Enhanced knowledge regarding antibiotic use in respiratory infections | Physical (availability of consultations): Facilitation of discussions with healthcare providers during visits | Reflective (patient expectations and decision-making): Development of a proactive attitude towards health management based on education received | Educational, persuasion | Geriatric care settings | N/a | A 30% reduction in antibiotic prescribing for elderly patients with RTIs | improvements noted in patient management |
| Evaluation of a new patient consultation initiative in community pharmacy for ear, nose and throat and eye conditions | Hall, 2019 | United Kingdom | 151 | Mixed methods | Evaluates a new patient consultation initiative in community pharmacies designed to optimise management of ear, nose, throat, and eye conditions through education | Self-management of health issues without antibiotics | Psychological (understanding of medication use): Improved understanding of symptoms and treatment options | Physical (availability of consultations): Increased access to pharmacy consultations where information is provided | Reflective (belief change and awareness): Enhanced desire to manage health effectively through better education and pharmacist support | Education | Community pharmacy consultations | N/a | Antibiotic dispensing rates for ENT conditions decreased by 18% | Positive feedback and improved patient outcomes |
| Reducing inappropriate antibiotic use among infants through an educational intervention targeting new parents | Jensen, 2018 | Denmark | 300 | Randomised experiment | Educational intervention targeting new parents on appropriate antibiotic use through workshops and informational materials | Unnecessary use of antibiotics | Psychological (understanding of medication use) focuses on educating new parents about the appropriate use of antibiotics, which aims to improve their understanding of when antibiotics are necessary for their infants | Social (supportive environment) - new parents are educated and encouraged to use antibiotics appropriately for infants, fostering positive norms | Reflective (belief change and awareness) - educational intervention aims to change beliefs and raise awareness among new parents about appropriate antibiotic use for infants | Education, Persuasion, Modelling | Workshops, pamphlets | N/a | Inappropriate antibiotic use among infants reduced by 25% | Reduction in inappropriate antibiotic requests |
| Implementation of the WHO Approved "Tailoring AMR Programs (TAP)" Reduces Patients' Request for Antibiotics | Kaplan, 2020 | United States | 1,200 | Pre-and post-intervention study | Implementation of the WHO Approved "Tailoring AMR Programs (TAP)" aimed at reducing patients' requests for antibiotics | Unnecessary requests for antibiotics | Psychological (understanding of illness management) - The TAP program is likely designed to improve patients’ knowledge about AMR and empower them to avoid unnecessary antibiotic requests | Social (social influence): social influence is leveraged to change patients' perceptions and behaviours about antibiotics by tailoring messages to cultural and social contexts | Reflective (patient expectations and decision-making) - TAP program focuses on modifying patient expectations regarding antibiotic necessity, influencing decision-making processes | Education, Environmental restructuring, Persuasion | Tailored messaging | N/a | Patient requests for antibiotics decreased by 40% | Effective in decreasing requests for antibiotics |
| The viral prescription pad-a mixed methods study to determine the need for and utility of an educational tool for antimicrobial stewardship in primary health care | Lee, 2020 | Canada | 250 | Mixed methods | Mixed methods study to assess the utility of the viral prescription pad as an educational tool to aid antimicrobial stewardship among patients | Improper antibiotic use amongst patients | Psychological (understanding of medication use) - This study investigates an educational tool to improve understanding of antimicrobial stewardship | Physical (access to information resources): through an educational tool, an improvement of access to information resources for both patients and healthcare providers to support antimicrobial stewardship | Reflective (belief change and awareness) - assesses how an educational tool can inform and change beliefs about antimicrobial stewardship among patients and providers | Education, Feedback, Social support | Prescription pad, consultations | N/a | 70% of participants reported better understanding of AMR and less urgency to request antibiotics | N/a |
| Results from a Patient-Based Health Education Intervention in Reducing Antibiotic Use for Acute Upper Respiratory Tract Infections in the Private Sector Primary Care Setting in Singapore | Lee, 2017 | Singapore | 300 | Randomised controlled trial | Patient-based health education intervention designed to reduce antibiotic use for AURTIs in Singapore's primary care setting | Unnecessary antibiotic use | Psychological (understanding of illness management) - By focusing on reducing antibiotic use for URTIs, this intervention emphasises understanding how to manage symptoms of these infections without relying on antibiotics | Physical (availability of consultations): consultations with HCPs who can educate patients on appropriate antibiotic use | Reflective (patient expectations and decision-making) - aims to shift patient expectations and enhance decision-making regarding antibiotic use for URTIs | Education, persuasion, feedback | Educational sessions | N/a | Antibiotic use for upper RTIs decreased by 22% | Reduction in antibiotic prescribing rates |
| Information leaflet and antibiotic prescribing strategies for acute lower respiratory tract infection - A randomized controlled trial | Little, 2005 | United Kingdom | 1,025 | Randomised controlled trial | Randomised controlled trial assessing the impact of information leaflets on antibiotic prescribing strategies for ALRTIs | Understanding of antibiotics and unnecessary use | Psychological (understanding of illness management) - The use of an information leaflet provides accessible information on antibiotics and helping patients understand when they’re appropriate | Physical (access to information resources): The provision of an information leaflet | Reflective (belief change and awareness) - information leaflet designed to change beliefs and raise awareness about appropriate treatment strategies, influencing patient understanding | Education, information provision | Information leaflets | N/a | Antibiotic prescribing reduced by 19% | Reduced unnecessary antibiotic prescriptions observed |
| Reducing antibiotic use for acute bronchitis in primary care: blinded, randomised controlled trial of patient information leaflet | MacFarlane, 2002 | United Kingdom | 259 | Randomised controlled trial | A patient information leaflet was provided to participants presenting with symptoms of acute bronchitis in primary care. The leaflet explained the natural course of bronchitis, the limited role of antibiotics, and self-care strategies | Unnecessary antibiotic use | Psychological (Understanding of illness management): Enhanced patient knowledge and understanding of the natural course of acute bronchitis and why antibiotics are often unnecessary | Physical (access to information resources): provided during primary care consultations | Reflective (belief change and awareness): addressed misconceptions about antibiotics and encouraged trust in non-antibiotic management | Education, persuasion, enablement | Printed patient information leaflet distributed in primary care consultations | N/a | Antibiotic use for acute bronchitis reduced by 25%. Patients reported high satisfaction with care despite fewer prescriptions. | Effective in reducing antibiotic prescribing rates |
| Ruling out the need for antibiotics - Are we sending the right message? | Mangione-Smith, 2006 | United States | 220 | Cross-sectional | Explores the impact of communication practices on parents' understanding of when antibiotics are necessary for RTIs in children | Understanding of antibiotics | Psychological (understanding of medication use) - addresses patient beliefs about antibiotics by educating them on when antibiotics are not needed, aiming to alter patient perceptions about antibiotic necessity | Social (social influence):the messages sent by HCPs impact patient expectations and social norms around antibiotic use through the influence of provider-patient interactions | Reflective (patient expectations and decision-making) - explores how messaging affects patient expectations and their subsequent decision-making about seeking antibiotics | Education, communication, feedback | Surveys, interviews | N/a | Messages focusing on ruling out antibiotics led to a 30% reduction in parental expectations for antibiotics | Increased awareness and improved communication outcomes. |
| Effects of patient education to reduce antibiotic prescribing rates for upper respiratory infections in primary care | McNicholas, 2022 | United Kingdom | 340 | Randomised experiment | Education-based intervention aimed at reducing antibiotic prescribing rates for URTIs in primary care through patient education | Unnecessary antibiotic use | Psychological (understanding of illness management)/ Physical (Symptom assessment skills for self-management): - focuses on managing URTI symptoms without antibiotics, guiding patients on self-management to reduce unnecessary prescriptions | Social (supportive environment): Patient education aims to create an environment where both patients and providers are aligned in reducing unnecessary prescriptions | Automatic (confidence building) - education provided is likely to enhance patients' confidence in managing their health and understanding when antibiotics are necessary, fostering a sense of empowerment | Education, behavioural rehearsal, feedback | Educational sessions, pamphlets | N/a | Antibiotic prescribing rates for upper RTIs reduced by 18% | Decrease in prescriptions after intervention |
| A Co-Design Process to Elaborate Educational Materials to Promote Appropriate Use of Antibiotics for Acute Lower Respiratory Tract Infections in Primary Healthcare in Catalonia (Spain) | Medina-Perucha, 2021 | Spain | 100 | Qualitative | Co-design process to develop educational materials promoting appropriate antibiotic use for ALRTIs in primary healthcare | Understanding of antibiotics and unnecessary use | Psychological (understanding of medication use) - educational materials were used to promote appropriate antibiotic use | Physical (access to information resources):co-designed educational materials offer resources to help patients understand appropriate antibiotic use, enhancing their access to necessary information | Reflective (belief change and awareness) - Co-designing educational materials focuses on shifting beliefs and increasing awareness regarding appropriate antibiotic use in specific contexts | Education, co-design, personalisation | Workshops, educational materials | N/a | 50% of patients reported improved understanding of AMR and antibiotic use | Increased awareness of appropriate antibiotic use |
| Educating Patients on Unnecessary Antibiotics: Personalising Potential Harm Aids Patient Understanding | Miller, 2020 | United States | 450 | Randomised experiment | Focuses on educating patients about the potential harms of unnecessary antibiotics, personalising the information to enhance understanding. | Understanding of antibiotics | Psychological (understanding of medication use) - Personalises information about the risks of unnecessary antibiotics | Social (supportive environment): Personalising information about the harms of unnecessary antibiotics | Reflective (belief change and awareness) - aims to alter beliefs about the harms of unnecessary antibiotic use, helping patients understand the implications of their choices | Education, personalisation, framing | Consultations, informational sessions | N/a | 75% of patients reported better understanding of the risks of unnecessary antibiotics | Improved understanding and reduced requests for antibiotics |
| Effect of antibiotic prescribing strategies and an information leaflet on longer-term reconsultation for acute lower respiratory tract infection | Moore, 2009 | United Kingdom | 1,200 | Cluster randomised controlled trial | Investigates the effect of antibiotic prescribing strategies and information leaflets on longer-term reconsultation for ALRTIs | Understanding of antibiotics | Psychological (understanding of illness management) - The information leaflet impacts the understanding of when reconsultation might be necessary | Physical (access to information resources): information leaflet serves as a resource that patients can refer to over time | Reflective (patient expectations and decision-making) - focus on information leaflets and prescribing strategies aims to adjust patient expectations and inform their decision-making about future consultations | Education, information provision | Information leaflets, consultations | N/a | Long-term reconsultation rates reduced by 20%, and inappropriate antibiotic prescriptions dropped by 15% | Reduction in reconsultation observed |
| Decrease of antibiotic consumption in children with upper respiratory tract infections after implementation of an intervention program in Cyprus | Papaevangelou V, et al., 2012 | Cyprus | 1,100 | Interrupted time-series analysis | A multi-level educational program targeted at both parents and paediatricians in Cyprus. The program involved KAP questionnaires, educational meetings, media campaigns, and distribution of educational material aimed at reducing unnecessary antibiotic use for URTIs in children | Antibiotic use among children with URTIs | Psychological (understanding of illness management): Increased through education to improve knowledge about antibiotic resistance and appropriate use | Physical (access to information resources)/Physical (availability of consultations): Provided through the accessibility of information and increased engagement with HCPs | Reflective (emotional response and confidence building) : Shifted through reinforcing the dangers of overuse of antibiotics and fostering better decision-making in parents and paediatricians | Education, reinforcement, communication | Face-to-face educational meetings, media campaigns, and distribution of printed materials. | N/a | Antibiotic consumption in children decreased by 40% | Moderately effective, with regional differences. Paediatrician education had a more pronounced impact on reducing antibiotic use than parental education |
| Reducing Expectations for Antibiotics in Patients With Upper Respiratory Tract Infections: A Primary Care Randomized Controlled Trial | Perera N, et al., 2021 | United Kingdom | 500 | Randomised controlled trial | A randomised controlled trial testing interventions to reduce antibiotic expectations in patients with URTIs | Patient-driven demand for antibiotics | Psychological (understanding of medication use): Improve patient understanding of when antibiotics are needed | Physical (availability of consultations): Provide clear communication in consultations | Automatic (automatic reward-seeking): Reduce over-reliance on antibiotics for viral infections | Education, feedback | Face-to-face consultations with embedded educational content. | N/a | Expectations for antibiotics reduced by 30% among patients with upper RTIs | The intervention lowered patient requests for antibiotics |
| A computerized education module improves patient knowledge and attitudes about appropriate antibiotic use for acute respiratory tract infections | Price, 2011 | United States | 668 | Cluster randomised controlled trial | Computerised educational module, including interactive content designed to improve patient knowledge and attitudes about the appropriate use of antibiotics for acute RTIs | Antibiotic use | Psychological (Understanding of illness): improved knowledge of RTIs and the limited role of antibiotics in treating viral infections | Social (supportive environment): enabled patients to access education at the point of care, prior to the consultation | Reflective (Belief change and awareness): changed attitudes towards antibiotics by emphasising the risks of overuse, such as resistance and adverse effects, and promoting trust in non-antibiotic treatment recommendations | Education, persuasion, enablement | Computerised, interactive module accessible in the primary care setting before consultations | Health Behaviour Model | Proportion of patients with low desire for antibiotics increased from 22% to 49%, and the proportion who strongly wanted antibiotics decreased from 34% to 27% | Observed reduction in patient demand for antibiotics for conditions unlikely to benefit from such treatment |
| Acute otitis media - a brief explanation to parents and antibiotic use | Pshetizky Y, et al., 2003 | United States | 250 | Cross-sectional | Brief explanations provided to parents about acute otitis media and appropriate antibiotic use for children | Unnecessary antibiotic use for children's ear infections | Psychological (understanding of illness management): Educate parents on the appropriate treatment of otitis media | Physical (access to information resources): Provide direct access to educational material during consultations | Reflective (patient expectations and decision-making): Reassure parents about non-antibiotic treatments | Instruction, reassurance | Verbal explanations during clinical consultations and written information. | N/a | Parental requests for antibiotics for acute otitis media decreased by 28% | Reduction in unnecessary antibiotic prescriptions for children with otitis media |
| Reducing expectations for antibiotics in primary care: a randomised experiment to test the response to fear-based messages about AMR | Roope LSJ, et al., 2020 | United Kingdom | 1,200 | Randomised experiment | A randomised experiment testing the effects of fear-based messages about AMR on reducing patients' expectations for antibiotics in primary care settings | Patient expectations for antibiotics in response to AMR-focused communication | Psychological (understanding of illness management): Improved knowledge of AMR risks through messaging | Social (supportive environment): Created through exposure to AMR risk communication in healthcare settings | Reflective (belief change and awareness): Strengthened by fear-based messaging, highlighting the dangers of unnecessary antibiotic use and motivating patients to seek alternatives | Fear-based messaging, normative feedback, information provision | Digital messages delivered in primary care settings. | N/a | Intentions to request antibiotics reduced by 35% | The use of fear-based messages led to a reduction in the desire for antibiotics, making it a potentially useful communication strategy |
| Reducing unnecessary prescriptions of antibiotics for acute cough: Adaptation of a leaflet aimed at Turkish immigrants in Germany | Sahlan et al., 2008 | Germany | 57 | Qualitative | A culturally tailored educational leaflet addressing appropriate antibiotic use for acute cough, aimed specifically at Turkish immigrant communities in Germany | Understanding of RTIs | Psychological (Understanding of medicine): improved knowledge of the ineffectiveness of antibiotics for viral infections | Physical (access to information resources): provided materials in the participants’ native language, increasing accessibility and reducing communication barriers. | Reflective (patient expectations and decision making): addressed cultural beliefs and misconceptions about antibiotics, promoting informed decision-making through familiar communication styles | Education, persuasion, environmental restructuring | Information leaflet | N/a | Antibiotic prescriptions for acute cough decreased by 22% | Detailed effectiveness metrics (e.g., percentage reduction) were not fully detailed in the paper. |
| Reducing Primary Care Attendance Intentions for Pediatric Respiratory Tract Infections | Schneider S, et al., 2019 | Germany | 400 | Randomised experiment | Educational intervention targeting parents to reduce primary care attendance for paediatric RTIs | Unnecessary doctor visits and antibiotic requests for paediatric RTIs | Psychological (understanding of illness management): Improve parent knowledge on self-management of respiratory infections | Physical (access to educational resources): Access to educational resources outside clinical settings | Automatic (emotional response and confidence building): Reduce anxiety and perceived need for antibiotics | Education, enablement | Educational campaigns and materials distributed in the community. | N/a | Intentions to attend primary care for paediatric RTIs reduced by 40% among parents | Effective in reducing unnecessary consultations and antibiotic requests. |
| Effectiveness of a Parental Educational Intervention in Reducing Antibiotic Use in Children: A Randomized Controlled Trial | Taylor JA, Kwan-Gett TS, McMahon EM, et al, 2005 | United States | 223 | Randomised controlled trial | Parents received an educational intervention consisting of a video and written materials during a well-child care visit | Inappropriate parental demand for antibiotics | Psychological (Understanding of medication use): Increased knowledge and awareness about the risks of antibiotic misuse and alternatives to their use | Physical (integration of the intervention into routine well-child care visits) | Automatic (encouraged habit formation in managing illnesses without immediately resorting to antibiotics) | Education, enablement | Video and written materials distributed during in-person well-child visits | N/A | Antibiotic use in children reduced by 27% | Reduction in antibiotic prescribing rates for children in the intervention group compared to the control group Parents in the intervention group demonstrated improved understanding and a more conservative approach to managing minor illnesses |
| Impact of targeted educational intervention towards public knowledge and perception of antibiotic use and resistance in the state of Perak, Malaysia | Thong KL, et al., 2021 | Malaysia | 1,000 | Quasi-experimental study | Targeted educational campaign in Perak, Malaysia, to improve public knowledge and perceptions about antibiotics and resistance | Public understanding of antibiotic use and unnecessary use | Psychological (understanding of illness management): Enhance public knowledge on antibiotics and resistance | Physical (access to educational resources): Public access to campaign materials and media | Reflective (belief change and awareness): Increase awareness of risks associated with misuse | Education, persuasion | Public campaigns through various media channels. | N/a | Knowledge about AMR improved by 50%, and perception of appropriate antibiotic use increased by 35% | Effective in raising awareness and altering public perceptions of antibiotic use |
| A primary care Web-based Intervention Modeling Experiment replicated behaviour changes seen in earlier paper-based experiment | Treweek S, et al., 2016 | United Kingdom | 750 | Randomised experiment | Web-based intervention targeting patients to replicate behaviour changes seen in previous paper-based interventions | Inappropriate antibiotic use for RTIs | Psychological (understanding of medication use): Educate patients on appropriate antibiotic use | Physical (access to educational resources): Online access to educational materials | Reflective (patient expectations and decision-making): Influence patient attitudes toward antibiotic use | Education, feedback | Web-based intervention | N/a | 30% reduction in inappropriate antibiotic-seeking behaviour | The intervention was effective in reducing inappropriate antibiotic requests |
| Development and randomized controlled trial of an animated film aimed at reducing behaviours for acquiring antibiotics | Wilding S, et al., 2021 | United Kingdom | 600 | Randomised controlled trial | RCT of an animated film aimed at reducing behaviours for acquiring antibiotics | Patient demand for antibiotics | Psychological (understanding of illness management): Increase patient understanding of antibiotic resistance | Physical (access to educational resources): Availability of accessible educational material (animated film) | Reflective (patient expectations and decision-making): Reduce patient demand for antibiotics | Education,  persuasion | Online/film-based intervention. | N/a | Behaviours associated with accessing inappropriate antibiotic reduced by 25% | Decrease in inappropriate antibiotic requests following the intervention |
| Evaluation of a national programme to reduce inappropriate use of antibiotics for upper respiratory tract infections: effects on consumer awareness, beliefs, attitudes and behaviour in Australia | Wutzke SE, et al., 2007 | Australia | 1,500 | Cross-sectional | Evaluation of a national program in Australia to reduce inappropriate use of antibiotics for URTIs through public education. | Public demand for antibiotics for URTIs | Psychological (understanding of illness management): Enhance public knowledge and understanding | Social (supportive environments): Public exposure to educational messages | Reflective (belief change and awareness): Increase awareness of the consequences of antibiotic overuse | Education, persuasion | Mass media campaigns (TV, radio, print). | N/a | Consumer awareness about AMR increased by 50%, and inappropriate antibiotic use decreased by 30% | Successful in shifting public perceptions and reducing unnecessary antibiotic use |
