# Supplementary material for: Behaviour change interventions addressing patient antibiotic treatment-seeking behaviour for respiratory tract infections in primary and community care settings: a scoping review
Source: BMJ Open. 2025 Aug 5;15(8):e101694. doi: 10.1136/bmjopen-2025-101694 (PMC12336482; doi:10.1136/bmjopen-2025-101694)
Supplement: Supplementary file 4 [file bmjopen-15-8-s003.docx]

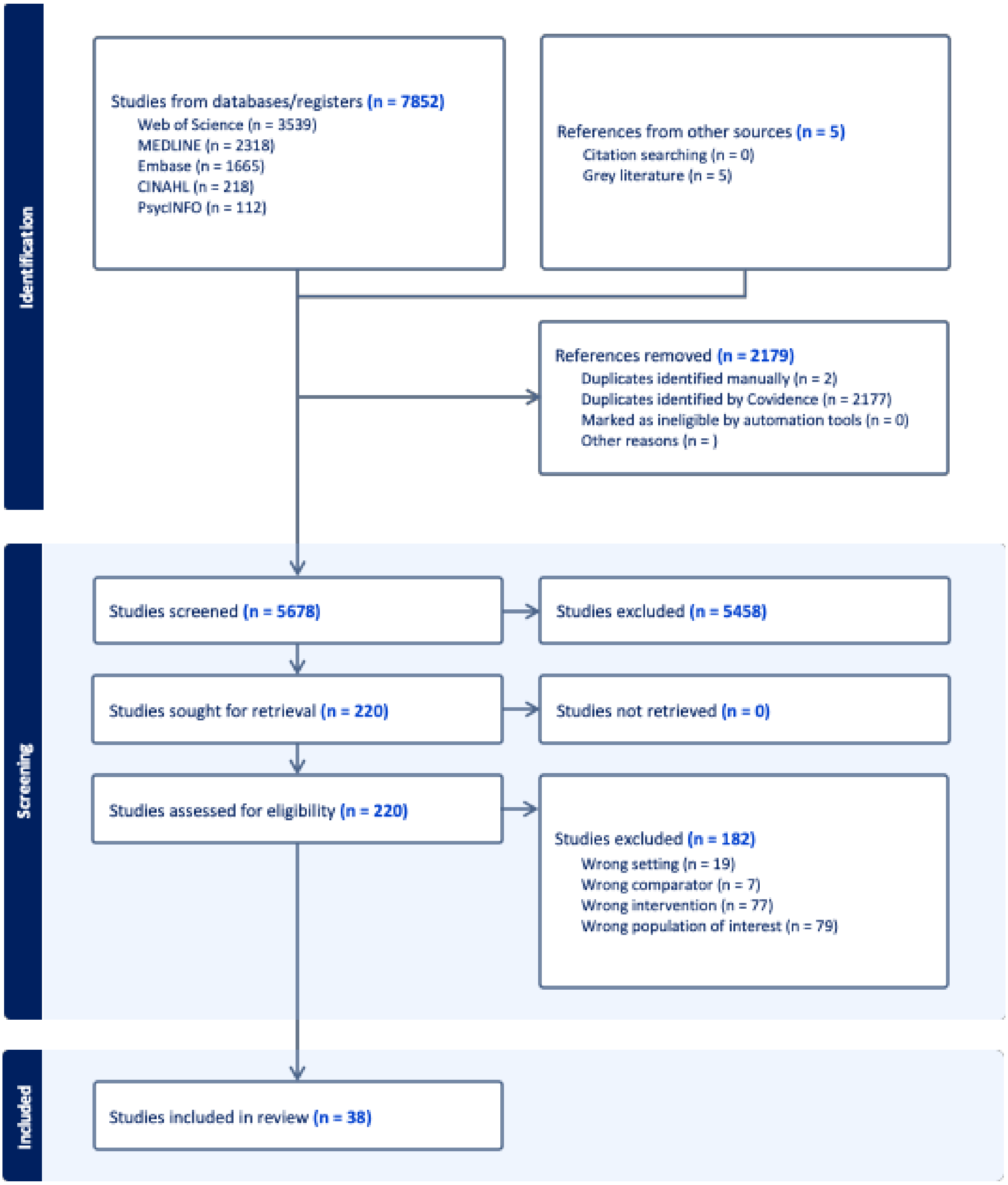
Identification
Studies from databases/registers (n = 7852)
Web of Science (n = 3539)
MEDLINE (n = 2318)
Embase (n = 1665)
CINAHL (n = 218)
PsycINFO (n = 112)
References from other sources (n = 5)
Citation searching (n = 0)
Grey literature (n = 5)
References removed (n = 2179)
Duplicates identified manually (n = 2)
Duplicates identified by Covidence (n = 2177)
Marked as ineligible by automation tools (n = 0)
Other reasons (n =)
Screening
Studies screened (n = 5678)
Studies excluded (n = 5458)
Studies sought for retrieval (n = 220)
Studies not retrieved (n = 0)
Studies assessed for eligibility (n = 220)
Studies excluded (n = 182)
Wrong setting (n = 19)
Wrong comparator (n = 7)
Wrong intervention (n = 77)
Wrong population of interest (n = 79)
Included
Studies included in review (n = 38)
