# Supplementary material for: Behaviour change interventions addressing patient antibiotic treatment-seeking behaviour for respiratory tract infections in primary and community care settings: a scoping review
Source: BMJ Open. 2025 Aug 5;15(8):e101694. doi: 10.1136/bmjopen-2025-101694 (PMC12336482; doi:10.1136/bmjopen-2025-101694)
Supplement: Supplementary file 5 [file bmjopen-2025-101694.reviewer_comments.pdf]

PEER REVIEW HISTORY

BMJ Open publishes all reviews undertaken for accepted manuscripts. Reviewers are asked to complete a checklist review form and are provided with free text boxes to elaborate on their assessment. These free text comments are reproduced below.

ARTICLE DETAILS

Title (Provisional)

Behaviour change interventions addressing patient antibiotic treatment seeking behaviour for respiratory tract infections in primary and community care settings: A scoping review

Authors

Maher, Anthony; Roche, K; Morrissey, Eimear; Murphy, Andrew; Sheaf, Greg; Ryan, Cristín; Molloy, Gerard J.

VERSION 1 - REVIEW

**Reviewer**	**1**
**Name**	**Godman, Brian**
**Affiliation**	**University of Strathclyde**
**Date**	**14-Apr-2025**
**COI**	**None**

Thank you for this paper.

I was very much looking forward to reading it because the pressure that patients place on prescribers (including nurses) in LMICs as well as dispensers to prescribe/ dispense antibiotics (including purchasing these without a prescription) often for self-limiting infections including acute respiratory infections, UTIs, acute diarrhoea and fevers - are a key reason for high rates of inappropriate use of antibiotics currently seen in LMICs (where ambulatory/ primary care accounts for up to 95% of antibiotic use in humans - Duffy E et al. Antibacterials dispensed in the community comprise 85%-95% of total human antibacterial consumption. J Clin Pharm Ther. 2018;43:59-64) and the associated high rates of AMR in these countries. Consequently, it is critical in these countries to address these issues and concerns if high and growing rates of AMR are to be reversed (especially following the ambitious goals for AMR reduction set out following the United Nations General Assembly on AMR in September 2024) - especially with rates of AMR appreciably higher in LMICs such as sub-Saharan African countries and Asian countries such as India, Pakistan, and Vietnam, than seen in high-income countries. High inappropriate usage of antibiotics in LMICs is exacerbated by often limited knowledge regarding antibiotics, AMR and AMS among

patients/ carers across LMICs (as well as often prescribers and dispensers). The situation is not helped by often language concerns where terms such as antibiotics/ AMR do not exist in a number of languages - so explanations need to be provided if patients are to take on board any instructions, etc., to help change their future behaviour. These issues, and their implications, are discussed in e.g. Saleem Z et al. Current access, availability and use of antibiotics in primary care among key low- and middle-income countries and the policy implications. Expert Rev Anti Infect Ther. 2025; Saleem Z et al. Status and implications of the knowledge, attitudes and practices towards AWaRe antibiotic use, resistance and stewardship among low- and middle-income countries. JAC-Antimicrobial Resistance. 2025;7:2; Sono TM et al. Pilot Study to Evaluate Patients' Understanding of Key Terms and Aspects of Antimicrobial Use in a Rural Province in South Africa Findings and Implications. Advances in Human Biology. 2025;15:108-12 and Sono et al. The Role of Patients with Addressing Inappropriate Dispensing of Antibiotics without a Prescription Especially in Developing Countries. Advances in Human Biology. 2025;15:1-4.

I was also disappointed to see only one paper in LMICs quoted - alongside a number of very old papers - although these may still be relevant. There was also no real mention of the appreciable differences that can exist between HICs and LMICs in terms of available resources/ personnel needed to undertake such practices in LMICs - although we are seeing some recent activities taking place to educate patients/ and their impact in LMICs, e.g. broader in Kandeel A et al. An educational intervention to promote appropriate antibiotic use for acute respiratory infections in a district in Egypt- pilot study. BMC Public Health. 2019;19:498; Maarouf L et al. Knowledge, attitudes and behaviour of Egyptians towards antibiotic use in the community: can we do better? Antimicrob Resist Infect Control. 2023;12:50; Aika IN et al. Bridging the gap in knowledge and use of antibiotics among pediatric caregivers: comparing two educational interventions. J Pharm Policy Pract. 2023;16:76; Tamhankar AJ et al. Characteristics of a Nationwide Voluntary Antibiotic Resistance Awareness Campaign in India; Future Paths and Pointers for Resource Limited Settings/Low and Middle Income Countries. Int J Environ Res Public Health. 2019;16; Herawati F, Yulia R, Arifin B, Frasetyo I, Setiasih, Woerdenbag HJ, et al. Educational Video Improves Knowledge about Outpatients' Usage of Antibiotics in Two Public Hospitals in Indonesia. Antibiotics. 2021;10 and Thong KS et al. Impact of targeted educational intervention towards public knowledge and perception of antibiotic use and resistance in the state of Perak, Malaysia. Antimicrob Resist Infect Control. 2021;10:29.

I can understand why you focused on ARIs as these are very prevalent conditions accounting for inappropriate use of antibiotics (in e.g. Godman B et al. Ongoing strategies to improve the management of upper respiratory tract infections and reduce inappropriate antibiotic use particularly among lower and middle-income countries: findings and implications for the future. Curr Med Res Opin. 2020;36:301-27.) - however by restricting your terms - this may account for the limited availability of papers in LMICs in your review.

Consequently - I would suggest some comments around these key issues in the Discussion especially if you want your paper to have utility among key stakeholder groups in critical LMICs - where the real issues/ challenges currently are to change behaviour/ reduce inappropriate prescribing and dispensing of antibiotics rather than high income countries such as Canada, Germany and the UK.

**Reviewer**	**2**
**Name**	**Clarke, Rebecca**
**Affiliation**	**University of Bristol, School of Psychological Science**
**Date**	**08-May-2025**
**COI**	**None**

A very timely, interesting and well-written review given the importance of AMR in healthcare. The authors present strong rationale for why this review is relevant. The recognition of multifaceted determinants influencing patients seeking antibiotics, the application of a behavioural framework, and conclusion that diverse intervention content is needed all represent valuable insights. This review will be of interest to many working within the field, both as practitioners and researchers.

With some minor revisions, I recommend publication.

Introduction –

1. Page 4, line 13-14. Please clarify the phrase "Positive influences on antibiotic prescribing" - I presume this refers to factors that reduce inappropriate antibiotic prescribing.

2. Page 4, line 23-24. The “negative connotations” mentioned are unclear.

Methodology –

3. Page 5- While participant types are specified in the inclusion criteria, study design inclusion criteria is not addressed. Were there any exclusion criteria based on study design?

Review findings –

4. Page 14 – “Intervention types” section. To strengthen statements such as "demonstrated that education addresses unnecessary antibiotic prescriptions," please either include supporting evidence, or reference specific parts of the table. Alternatively, this section is about “intervention types”, not intervention effectiveness. In which case, should intervention effectiveness be included here?

5. Page 15, line 3. “reduced inappropriate behaviours” – please specify what the inappropriate behaviours are.

6. Page 15, lines 3-6. For consistency with the other statements, consider including percentages or statistics with “Behavioural outcomes encompassed reductions in antibiotic prescriptions and instances of self-medication in four studies.”

VERSION 1 - AUTHOR RESPONSE

Reviewer: 1
Dr. Brian Godman, University of Strathclyde
Comments to the Author:
Thank you for this paper.

I was very much looking forward to reading it because the pressure that patients place on prescribers (including nurses) in LMICs as well as dispensers to prescribe/ dispense antibiotics (including purchasing these without a prescription) often for self-limiting infections including acute respiratory infections, UTIs, acute diarrhoea and fevers - are a key reason for high rates of inappropriate use of antibiotics currently seen in LMICs (where ambulatory/ primary care accounts for up to 95% of antibiotic use in humans - Duffy E et al. Antibacterials dispensed in the community comprise 85%-95% of total human antibacterial consumption. J Clin Pharm Ther. 2018;43:59-64) and the associated high rates of AMR in these countries. Consequently, it is critical in these countries to address these issues and concerns if high and growing rates of AMR are to be reversed (especially following the ambitious goals for AMR reduction set out following the United Nations General Assembly on AMR in September 2024) - especially with rates of AMR appreciably higher in LMICs such as sub-Saharan African countries and Asian countries such as India, Pakistan, and Vietnam, than seen in high-income countries. High inappropriate usage of antibiotics in LMICs is exacerbated by often limited knowledge regarding antibiotics, AMR and AMS among patients/ carers across LMICs (as well as often prescribers and dispensers). The situation is not helped by often language concerns where terms such as antibiotics/ AMR do not exist in a number of languages - so explanations need to be provided if patients are to take on board any instructions, etc., to help change their future behaviour. These issues, and their implications, are discussed in e.g. Saleem Z et al. Current access, availability and use of antibiotics in primary care among key low- and middle-income countries and the policy implications. Expert Rev Anti Infect Ther. 2025; Saleem Z et al. Status and implications of the knowledge, attitudes and practices towards AWaRe antibiotic use, resistance and stewardship among low- and middle-income countries. JAC-Antimicrobial Resistance. 2025;7:2; Sono TM et al. Pilot Study to Evaluate Patients’ Understanding of Key Terms and Aspects of Antimicrobial Use in a Rural Province in South Africa Findings and Implications. Advances in Human Biology. 2025;15:108-12 and Sono et al. The Role of Patients with Addressing Inappropriate Dispensing of Antibiotics without a Prescription Especially in Developing Countries. Advances in Human Biology. 2025;15:1-4.

I was also disappointed to see only one paper in LMICs quoted - alongside a number of very old papers - although these may still be relevant. There was also no real mention of the appreciable differences that can exist between HICs and LMICs in

terms of available resources/ personnel needed to undertake such practices in LMICs - although we are seeing some recent activities taking place to educate patients/ and their impact in LMICs, e.g. broader in Kandeel A et al. An educational intervention to promote appropriate antibiotic use for acute respiratory infections in a district in Egypt- pilot study. BMC Public Health. 2019;19:498; Maarouf L et al. Knowledge, attitudes and behaviour of Egyptians towards antibiotic use in the community: can we do better? Antimicrob Resist Infect Control. 2023;12:50; Aika IN et al. Bridging the gap in knowledge and use of antibiotics among pediatric caregivers: comparing two educational interventions. J Pharm Policy Pract. 2023;16:76; Tamhankar AJ et al. Characteristics of a Nationwide Voluntary Antibiotic Resistance Awareness Campaign in India; Future Paths and Pointers for Resource Limited Settings/Low and Middle Income Countries. Int J Environ Res Public Health. 2019;16; Herawati F, Yulia R, Arifin B, Frasetyo I, Setiasih, Woerdenbag HJ, et al. Educational Video Improves Knowledge about Outpatients' Usage of Antibiotics in Two Public Hospitals in Indonesia. Antibiotics. 2021;10 and Thong KS et al. Impact of targeted educational intervention towards public knowledge and perception of antibiotic use and resistance in the state of Perak, Malaysia. Antimicrob Resist Infect Control. 2021;10:29.

I can understand why you focused on ARIs as these are very prevalent conditions accounting for inappropriate use of antibiotics (in e.g. Godman B et al. Ongoing strategies to improve the management of upper respiratory tract infections and reduce inappropriate antibiotic use particularly among lower and middle-income countries: findings and implications for the future. Curr Med Res Opin. 2020;36:301-27.) - however by restricting your terms - this may account for the limited availability of papers in LMICs in your review.

Consequently - I would suggest some comments around these key issues in the Discussion especially if you want your paper to have utility among key stakeholder groups in critical LMICs - where the real issues/ challenges currently are to change behaviour/ reduce inappropriate prescribing and dispensing of antibiotics rather than high income countries such as Canada, Germany and the UK.

Response: We agree that inappropriate use of antibiotics in LMIC contexts is a major global health challenge for AMR. The programme of work in which this systematic review is embedded is a PhD focused on antibiotic use in a HIC context namely Republic of Ireland, therefore there is a focus on common health conditions i.e. URTIs and inappropriate use of antibiotics on this and similar socio-cultural contexts. In response to your helpful feedback, we have now added the following paragraph to the discussion to acknowledge the limitations of this socio-cultural focus.

“The majority of included studies in this review were carried out in high-income countries (HICs) where the prevailing population health challenges and health services limitations are very different to low- and middle-income countries (LMICs). For example, there are typically significantly different patterns of infectious diseases and associated acute illnesses e.g. diarrhoea and fever, and there can be a shortage or complete absence of trained prescribers of antibiotics and public health resourcing (1,2). In addition, there can be limitations in population health literacy and a diversity of informal health communication channels, which mean that the causes and communication solutions to unnecessary use of antibiotics are likely to be a different

form than HIC contexts.(3–5) Therefore, behaviour change interventions need to factor in the appreciable differences that can exist between HICs and LMICs in terms of available resources and personnel needed to implement any behaviour change interventions to reduce inappropriate use of antibiotics.”

1. Walker CLF, Rudan I, Liu L, Nair H, Theodoratou E, Bhutta ZA, et al. Global burden of childhood pneumonia and diarrhoea. The Lancet. 2013 Apr;381(9875):1405–16.
2. Troeger C, Blacker BF, Khalil IA, Rao PC, Cao S, Zimsen SR, et al. Estimates of the global, regional, and national morbidity, mortality, and aetiologies of diarrhoea in 195 countries: a systematic analysis for the Global Burden of Disease Study 2016. Lancet Infect Dis. 2018 Nov;18(11):1211–28.
3. Pearson M, Chandler C. Knowing antimicrobial resistance in practice: a multi-country qualitative study with human and animal healthcare professionals. Glob Health Action. 2019 Dec 13;12(sup1):1599560.
4. Ocan M, Obuku EA, Bwanga F, Akena D, Richard S, Ogwal-Okeng J, et al. Household antimicrobial self-medication: a systematic review and meta-analysis of the burden, risk factors and outcomes in developing countries. BMC Public Health. 2015 Dec;15(1):742.
5. Auta A, Hadi MA, Oga E, Adewuyi EO, Abdu-Aguye SN, Adeloye D, et al. Global access to antibiotics without prescription in community pharmacies: A systematic review and meta-analysis. J Infect. 2019 Jan;78(1):8–18.

Reviewer: 2
Dr. Rebecca Clarke, University of Bristol
Comments to the Author:
A very timely, interesting and well-written review given the importance of AMR in healthcare. The authors present strong rationale for why this review is relevant. The recognition of multifaceted determinants influencing patients seeking antibiotics, the application of a behavioural framework, and conclusion that diverse intervention content is needed all represent valuable insights. This review will be of interest to many working within the field, both as practitioners and researchers.

With some minor revisions, I recommend publication.

Introduction –
1. Page 4, line 13-14. Please clarify the phrase "Positive influences on antibiotic prescribing" - I presume this refers to factors that reduce inappropriate antibiotic prescribing.
Thank you for this clarification request. We have revised the text to clarify that “positive influences” refers specifically to factors that support appropriate antibiotic prescribing practices and reduce instances of unnecessary prescribing.

2. Page 4, line 23-24. The “negative connotations” mentioned are unclear.
We appreciate the reviewer pointing this out to us. We have revised this section to clarify what is meant by “negative connotations,” specifying that it refers to perceptions among clinicians that stewardship efforts may imply criticism of their clinical judgment or undermine patient-provider trust.

Methodology –
3. Page 5- While participant types are specified in the inclusion criteria, study design inclusion criteria is not addressed. Were there any exclusion criteria based on study design?
Thank you for highlighting this omission. We have now added a sentence to explicitly state the inclusion and exclusion criteria based on study design.

Review findings –
4. Page 14 – "Intervention types" section. To strengthen statements such as "demonstrated that education addresses unnecessary antibiotic prescriptions," please either include supporting evidence, or reference specific parts of the table. Alternatively, this section is about "intervention types", not intervention effectiveness. In which case, should intervention effectiveness be included here?
We appreciate this point. To maintain clarity in the "Intervention types" section, we have revised the wording to avoid making claims about effectiveness unless specifically supported by the data. Effectiveness outcomes are now primarily reported in the subsequent section "outcome measures and effectiveness".

5. Page 15, line 3. "reduced inappropriate behaviours" – please specify what the inappropriate behaviours are.
Thank you for pointing out the need for specificity. We have revised the sentence to specify that "inappropriate behaviours" refers to self-medicating with antibiotics without a prescription, pressuring healthcare providers for antibiotics, or misusing antibiotics for viral infections.

6. Page 15, lines 3-6. For consistency with the other statements, consider including percentages or statistics with "Behavioural outcomes encompassed reductions in antibiotic prescriptions and instances of self-medication in four studies."
We appreciate this suggestion to improve consistency and precision. We have now added quantitative findings from the relevant studies to better illustrate the impact of interventions on behavioural outcomes such as reductions in self-medication and antibiotic requests.
